# The Effectiveness of Telerehabilitation in Managing Pain, Strength, and Balance in Adult Patients With Knee Osteoarthritis: Systematic Review

**DOI:** 10.2196/72466

**Published:** 2025-04-08

**Authors:** Theodora Plavoukou, Michail Iosifidis, Georgios Papagiannis, Dimitrios Stasinopoulos, Georgios Georgoudis

**Affiliations:** 1 University of West Attica Athens Greece

**Keywords:** telerehabilitation, knee osteoarthritis, pain management, remote physiotherapy, digital health, remote therapy, physiotherapy, pain management, strength, balance, functional mobility, rehabilitation technology

## Abstract

**Background:**

Knee osteoarthritis (KOA) is a chronic, degenerative joint disease characterized by pain, stiffness, and functional impairment, significantly affecting mobility and quality of life. Traditional rehabilitation, mainly through in-person physiotherapy, is widely recommended for KOA management. However, access to these services is often limited due to geographic, financial, and mobility constraints. Telerehabilitation has emerged as an alternative, providing remote rehabilitation through digital platforms. Despite its increasing adoption, its effectiveness in improving key functional parameters such as pain, strength, and balance remains uncertain. While previous studies have focused primarily on pain relief and overall functional improvement, a broader assessment of its impact on mobility and fall prevention is needed.

**Objective:**

This systematic review examines the effectiveness of telerehabilitation in improving pain, strength, and balance in adults with KOA compared with traditional rehabilitation or no intervention. In addition, it evaluates the impact of different telerehabilitation models, such as therapist-guided versus self-managed programs, and explores the feasibility of integrating telerehabilitation as an alternative in KOA management.

**Methods:**

A systematic search of 4 databases (PubMed, PEDro, Cochrane, and Scopus) was conducted to identify randomized controlled trials (RCTs) published from May 2004 to May 2024. Inclusion criteria consisted of adults with KOA, evaluation of telerehabilitation either as a stand-alone intervention or in comparison to traditional rehabilitation or no intervention, and measurement of at least one primary outcome (pain, strength, or balance). A total of 2 independent reviewers assessed the risk of bias using validated tools. Due to variations in intervention programs and assessment methods, a narrative synthesis was performed instead of a meta-analysis. The review followed established guidelines, and data extraction was conducted using appropriate software.

**Results:**

A total of 6 RCTs (N=581 participants) met the inclusion criteria. The results indicate that telerehabilitation effectively reduces pain and improves strength and balance, although the extent of benefits varies. Some studies reported similar pain reductions between telerehabilitation and traditional rehabilitation, while others highlighted greater functional improvements in telerehabilitation groups. Therapist-guided telerehabilitation was associated with higher adherence rates and better functional outcomes compared with self-managed programs. The risk of bias assessment showed that most studies were of moderate to good quality, though common issues included selection bias, performance bias, and participant attrition.

**Conclusions:**

Telerehabilitation is a promising alternative for KOA management, especially for individuals facing barriers to in-person therapy. It is effective in reducing pain and improving strength and balance, though its success depends on patient engagement, intervention delivery, and rehabilitation protocols. Therapist-guided programs yield better outcomes than self-managed approaches. Further research is needed to standardize intervention protocols, integrate emerging technologies, and evaluate cost-effectiveness to guide clinical practice and health care policies.

**Trial Registration:**

PROSPERO CRD42024564141; https://tinyurl.com/25ykvy7d

## Introduction

Knee osteoarthritis (KOA) is a chronic, progressive condition characterized by the degeneration of joint cartilage and subchondral bone, leading to pain, stiffness, and functional impairment [1]. It is one of the most prevalent forms of arthritis, significantly affecting millions of individuals worldwide, particularly older adults, and is a leading cause of disability [2]. Epidemiological data indicate that KOA disproportionately affects women, with an estimated prevalence of 10% in men and 13% in women ages 60 years and older [3]. The burden of KOA is expected to rise due to increased life expectancy and the growing obesity epidemic, both of which are major contributors to the disease’s progression [3]. Despite the availability of various treatment strategies, including pharmacological interventions, physical therapy, and surgical options, accessibility and effectiveness remain substantial challenges, prompting the exploration of alternative therapeutic approaches [4].

Among conservative treatments, physical therapy with structured exercise programs is considered a cornerstone in KOA management. Evidence consistently supports its role in improving joint function, reducing pain, and enhancing overall quality of life [5]. However, access to traditional in-person rehabilitation is often restricted by geographic, financial, and mobility-related barriers, which prevent many patients from receiving the necessary care [6]. These limitations were further exacerbated by the COVID-19 pandemic, which significantly disrupted health care services and underscored the urgent need for innovative, remote solutions to ensure continuous patient care [7]. In response to these challenges, telerehabilitation has emerged as a viable alternative, leveraging digital technologies to deliver rehabilitation programs remotely.

Telerehabilitation encompasses a broad spectrum of services, including guided exercise programs, educational resources, and real-time feedback from health care providers through telecommunication platforms [8]. This approach offers several advantages, including improved accessibility, convenience, and the ability to provide continuous, personalized care, which is particularly beneficial for chronic conditions such as KOA [4]. Furthermore, telerehabilitation allows health care providers to monitor patient progress and modify treatment plans accordingly, ensuring a more tailored therapeutic experience [9]. Despite its potential, the integration of telerehabilitation into routine clinical practice has been slow, partly due to uncertainties regarding its effectiveness compared to conventional in-person rehabilitation.

Current evidence on telerehabilitation for KOA includes several randomized controlled trials (RCTs) suggesting its benefits in improving pain, physical function, and patient-reported outcomes [1]. However, existing studies exhibit considerable heterogeneity in design, intervention protocols, and outcome measures, making it challenging to derive definitive conclusions regarding its overall efficacy. Previous systematic reviews, such as Xie et al [1], focused primarily on pain and functional improvements without incorporating other critical outcomes, such as strength and balance, which are essential for functional mobility and fall prevention in patients with KOA. Furthermore, past reviews have not comprehensively examined differences between telerehabilitation modalities, such as real-time therapist-guided interventions versus self-managed web-based programs, limiting the depth of analysis regarding intervention effectiveness.

This systematic review aims to address these gaps by synthesizing findings from RCTs that have investigated the impact of telerehabilitation on key clinical outcomes, including pain, strength, and balance, in adult patients with KOA. By expanding the scope beyond pain and function, this review provides a more comprehensive understanding of telerehabilitation’s role in enhancing musculoskeletal performance and reducing fall risk—factors crucial for long-term mobility and independence. In addition, this review includes an updated literature search extending to May 2024, incorporating 6 newly identified RCTs, thereby significantly expanding the available evidence base.

Another key differentiator of this review is its methodological rigor. Unlike previous reviews, this study applies dual risk-of-bias assessments using both the Physiotherapy Evidence Database (PEDro) scale and the Downs and Black checklist, ensuring a more robust evaluation of study quality. Furthermore, while past reviews often compared telerehabilitation to a single standard-of-care intervention, this review adopts a broader comparative framework, analyzing its effectiveness against multiple rehabilitation strategies, including clinic-based physiotherapy, home-based exercise programs, and no intervention groups. This approach allows for a more nuanced understanding of telerehabilitation’s place within the spectrum of KOA management strategies.

Initially, this review intended to conduct a meta-analysis; however, substantial heterogeneity across studies—stemming from differences in intervention protocols, outcome measures, and control groups—precluded a reliable quantitative synthesis. High statistical heterogeneity (*I*² values exceeding 50%) indicated that pooling results would not yield meaningful conclusions. Consequently, a narrative synthesis was chosen to provide a detailed qualitative comparison of the available evidence. This decision ensures that findings are interpreted within the appropriate clinical context rather than forcing an unreliable statistical synthesis.

The specific objectives of this review are 2-fold: first, to assess whether telerehabilitation is associated with significant improvements in pain, physical function, strength, and balance in patients with KOA compared with traditional therapy or usual care; and second, to determine whether different telerehabilitation modalities yield varying degrees of effectiveness in managing KOA symptoms. By addressing these questions, this review seeks to establish a clearer understanding of telerehabilitation’s role in KOA management and provide evidence-based recommendations for its integration into clinical practice.

In conclusion, as the prevalence of KOA continues to rise, so does the demand for accessible and effective rehabilitation strategies. Telerehabilitation represents a promising solution, offering remote, individualized, and scalable interventions for KOA management [4]. This systematic review will contribute to the growing body of literature by providing a comprehensive evaluation of telerehabilitation’s impact on pain, strength, and balance in adults with KOA, ultimately informing clinical decision-making and guiding the development of future therapeutic interventions.

## Methods

### Study Protocol and Registration

All analyses were based on data from previously published studies. Thus, no ethical approval or patient consent was required. The review was conducted according to the PRISMA (Preferred Reporting Items for Systematic Reviews and Meta-Analyses) statement (see PRISMA checklist in Multimedia Appendix 1) [10]. The a priori protocol for the review is published in the International Prospective Register of Systematic Reviews (PROSPERO; CRD42024564141).

### Information Sources

A search for RCTs was conducted across the databases PubMed, PEDro, Cochrane, and Scopus. A total of 207 studies were identified: 83 papers from PubMed, 10 from PEDro, 1 from Cochrane, and 113 from Scopus. The primary keywords used were telerehabilitation, pain management, strength, balance, and physical therapy. Only articles published in English were selected.

### Search and Eligibility Criteria

#### Overall Search Strategy

The search strategy was systematically developed by the research team following established systematic review guidelines. The search was conducted on May 1, 2024, across multiple electronic databases using a predefined combination of keywords: (osteoarthritis OR osteoarthrosis OR cartilage OR degenerative arthritis) AND (telemedicine OR e-health OR telehealth OR telerehabilitation OR internet OR web OR online) AND knee osteoarthritis. The exact search strategy, including Boolean operators, filters, and databases, has been provided as supplementary material.

In addition to the database search, manual screening of reference lists from the identified papers was conducted to ensure comprehensive literature coverage. The inclusion and exclusion criteria were determined based on the PICO (Population, Intervention, Comparison, Outcome) framework [11,12], ensuring methodological rigor and relevance.

#### Interventions

The studies included in this review evaluated the impact of internet-based rehabilitation programs in comparison with conventional rehabilitation approaches, such as in-clinic or hospital-based therapy, as well as no intervention (waiting list control). In the internet-based rehabilitation programs, therapy was either provided as a stand-alone treatment or in combination with traditional physiotherapy. These programs typically featured video demonstrations, graphical educational materials, real-time communication with health care providers (including physicians and therapists), and group discussions designed to support self-directed rehabilitation for individuals with KOA. The rehabilitation methods used encompassed a range of strategies, including structured exercises, patient education, and self-management techniques.

#### Outcome Measures

The primary outcome measures assessed in the included studies were pain, strength, and balance. These outcomes were evaluated using various validated assessment tools and aimed at determining the effectiveness of internet-based rehabilitation in improving these key functional parameters in individuals with knee OA.

#### Inclusion Criteria

For this systematic review, only RCTs were selected, with restrictions on the publication year (2004-2024). Participant characteristics were (1) adult patients with KOA, (2) types of interventions: the articles had to assess the effect of telerehabilitation either as a stand-alone therapy or in comparison with another intervention, and 30 outcome measures: the factors evaluated were pain and/or strength and/or balance.

#### Exclusion Criteria

The exclusion criteria included patients under the age of 18 years, studies without described protocols, incomplete papers, systematic reviews or meta-analyses, case studies, and papers written in languages other than English. We selected only RCTs as they provide the highest level of evidence by minimizing bias through randomization. This strengthens the validity of our findings and ensures methodological rigor. In addition, the PEDro scale and Modified Downs and Black checklist, used for quality assessment, are specifically designed for RCTs, further reinforcing the reliability of our review.

#### Data Extraction and Management

The papers for this systematic review were managed using the “RAYYAN” platform, where duplicate entries were initially removed. In the first stage, 2 independent reviewers, TP and MI, blindly screened the titles and abstracts of the papers and removed those deemed unsuitable. In the subsequent stage, the 2 reviewers examined the full texts of the articles based on the predefined inclusion and exclusion criteria. After identifying the appropriate RCTs, data extraction was performed.

#### Risk of Bias

In this systematic review, the PEDro scale and the Modified Downs and Black checklist were used to assess the risk of bias. A total of 2 independent reviewers, TP and MI, applied these tools and rated the RCTs. Any disagreements between the two reviewers were resolved through discussion or with the help of a third evaluator, GG.

## Results

### Search and Selection

**Figure 1:**
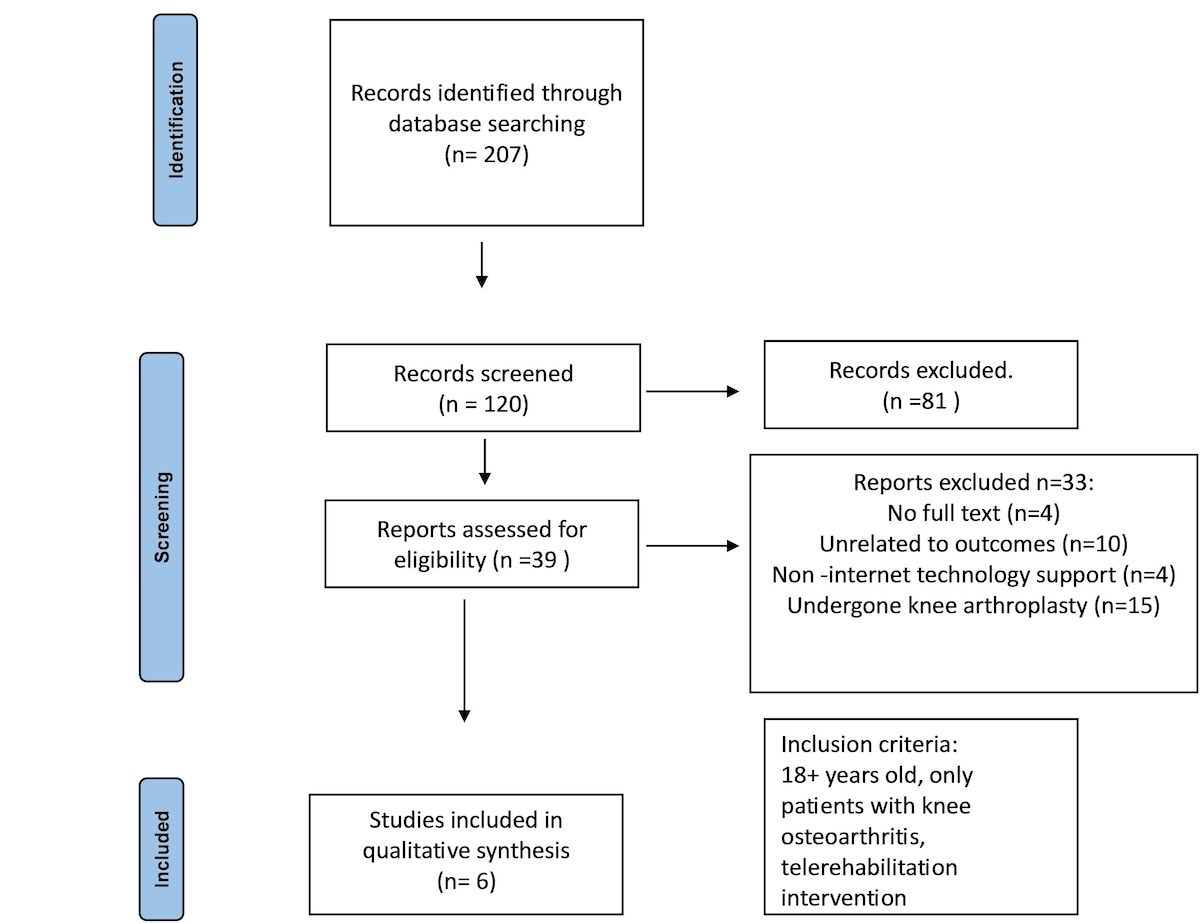
Flow chart of the search and selection process.

A systematic search was conducted across multiple databases, including PubMed (n=83), PEDro (n=10), Cochrane (n=1), and Scopus (n=113), identifying a total of 207 records (Figure 1). After removing duplicates, 120 records remained for screening, where 2 independent reviewers (TP and MI) assessed titles and abstracts using the “RAYYAN” platform. Following this process, 81 records were excluded, and 39 reports were assessed for eligibility. Of these, 33 reports were further excluded due to reasons such as the absence of full text (n=4), irrelevance to the outcome (n=10), noninternet technology support (n=4), or participants having undergone knee arthroplasty (n=15). Ultimately, 6 studies met the predefined inclusion criteria, involving a total of 581 patients with KOA who received telerehabilitation intervention. The inclusion criteria required participants to be at least 18 years old, diagnosed with KOA, and undergoing telerehabilitation intervention.

### Intervention Programs

The interventions in the 6 included RCTs were as follows: (1) telerehabilitation compared with electrotherapy and home exercise [9], (2) telerehabilitation through Zoom (Zoom Video Communications) compared with individual home rehabilitation with the help of a booklet [13], (3) telerehabilitation combined with a web-based PCST (pain coping skills training) rehabilitation program compared with usual care [14], (4) telerehabilitation compared with clinic-based rehabilitation [6], (5) telerehabilitation compared with home rehabilitation [15], and (6) telerehabilitation with an 8-week PAINCOACH (pain management coaching) program compared with rehabilitation without PAINCOACH [16]. In most cases, both groups in all the studies followed the same rehabilitation program, with the main difference being that the intervention group was always guided by a physiotherapist through telecommunication.

### Risk of Bias

For the PEDro scale, the reviewers applied the 11 criteria included in the tool and categorized the studies based on their total scores: 0-3 were considered “POOR,” 4-5 “FAIR,” 6-8 “GOOD,” and 9-10 “EXCELLENT.” The PEDro scale criteria are outlined in Table 1.

The above RCTs were evaluated using the PEDro scale [17]. The average score across the 6 studies was 6.3/10, indicating good quality. More specifically, 1 study scored 8/10 [16], 2 studies scored 7/10 [13,15], and 2 studies scored 6/10 [6,9], all of which are considered good quality according to the scale. However, 1 study scored only 4/10, which classifies it as a fair-quality study (Table 2) [14].

**Table 1:** Physiotherapy Evidence Database (PEDro) scale criteria.

RCT^a^	1	2	3	4	5	6	7	8	9	10	11	Score^b^	
Azma et al [9], 2017	+	+	–	+	–	–	–	+	+	+	+	6/10	
Tore et al [13], 2022	+	+	–	+	–	–	+	+	+	+	+	7/10	
Lawford et al [14], 2018	+	–	–	–	–	–	–	+	+	+	+	4/10	
Odole and Ojo [6], 2013	+	+	–	+	–	–	–	+	+	+	+	6/10	
Bennell et al [15], 2016	+	+	+	+	+	–	+	–	–	+	+	7/10	
Rini et al [16], 2015	+	+	+	+	–	+	–	+	+	+	+	8/10	
MO average													6.3/10

^a^RCT: randomized controlled trial.

^b^Average=6.3/10, green=excellent (9-10), blue=good (6-8), yellow=fair (4-5), and red=poor (0-3).

**Table 2:** Downs and Black scores.

RCT^a^	Downs and Black score^b^	Level of methodological quality
Azma et al [9], 2017	18/28	FAIR
Tore et al [13], 2022	20/28	GOOD
Lawford et al [14], 2018	16/28	FAIR
Odole and Ojo [6], 2013	19/28	FAIR
Bennell et al [15], 2016	19/28	FAIR
Rini et al [16], 2015	22/28	GOOD

^a^RCT: randomized controlled trial.

^b^RED=POOR (≤14), YELLOW=FAIR (15-19), BLUE=GOOD (20-25), GREEN=EXCELLENT (26-28).

Based on the Downs and Black checklist [18], 4 studies were classified as “FAIR” quality [6,9,14,15], and the remaining 2 studies were classified as “GOOD” quality [13,16].

### Limitations

Although the initial objective of this systematic review was to conduct a meta-analysis, significant heterogeneity among the included studies prevented a reliable quantitative synthesis. Variability in intervention protocols (eg, therapist-guided video calls, self-directed web-based programs, and mobile apps), diverse outcome measures (eg, Western Ontario and McMaster Universities Osteoarthritis Index [WOMAC], Visual Analog Scale [VAS], timed up and go test [TUG], and 30-second chair stand test [30-CST]), and differences in comparison groups (eg, traditional physiotherapy, no intervention, home-based exercises) introduced methodological inconsistencies. In addition, high statistical heterogeneity (*I*²>50%) further undermined the validity of a meta-analysis. As a result, a narrative synthesis was conducted to provide a qualitative comparison of findings.

### Study Characteristics

The baseline descriptive characteristics (country, sample size, age, and gender) of the 6 studies included in the systematic review are summarized in Table 3. A total of 2 studies were from Australia, 1 from the United States, 1 from Brazil and Iran, 1 from Turkey, and 1 from Nigeria. The mean age of patients with KOA ranged from 37 to 72 years, and all studies included both men and women.

**Table 3:** Summary of studies on telerehabilitation in knee osteoarthritis.

Study title	Authors	Sample size and condition	Assessment tools	Method	Outcome
Efficacy of telerehabilitation compared with office-based physical therapy in patients with knee osteoarthritis: A randomized clinical trial	Azma et al [9], 2017	Knee osteoarthritis (N=54)	VAS^a^ KOOS^b^ WOMAC^c^	Telerehabilitation group: 3 times/week for 6 weeks. Control group: 3 times/week in the clinic for 6 weeks.	Significant improvement in both groups on all scales, no significant differences between groups.
The quality of physiotherapy and rehabilitation programs and the effect of telerehabilitation on patients with knee osteoarthritis	Tore et al [13], 2022	Knee osteoarthritis (N=48)	30 CST^d^ KOOS NRS^e^ IPAQ-SF^f^ FSS^g^ QUIPA^h^ EARS^i^ HADS^j^ TKS^k^ PAR-Q+^l^	Telerehabilitation through Zoom for 8 weeks, 3 times per week (Group A). Control group exercised alone at home (Group B).	Group A showed significant improvement in strength, balance, and other parameters compared with group B.
Moderators of effects of internet-delivered exercise and pain coping skills training for people with knee osteoarthritis: exploratory analysis of the IMPACT randomized controlled trial	Lawford et al [14], 2018	Knee osteoarthritis (N=148)	NRS WOMAC	Intervention: Exercise and pain management training via Skype and on the web. Control: Educational materials only.	Significant pain reduction in employed individuals of the intervention group compared with the control group.
A telephone-based physiotherapy intervention for patients with osteoarthritis of the knee	Odole and Ojo et al [6], 2013	Knee osteoarthritis (N=50)	VAS IKHOAM^m^	Telerehabilitation with physiotherapist guidance 3 times/week for 6 weeks. Control group: clinic-based physiotherapy.	Significant improvement in both groups in pain and functionality, no significant differences between groups.
Telephone coaching to enhance a home-based physical activity program for knee osteoarthritis: a randomized clinical trial	Bennell et al [15], 2016	Knee osteoarthritis (N=168)	NRS WOMAC PACE^n^ AAS^o^ GROC^p^	Both groups received 5 individual physiotherapy sessions over 6 months. Group A received an additional 6 coaching sessions by telephone.	Significant improvement in pain and function for both groups, with Group A showing greater improvement in physical activity at 6 months.
Automated Internet-based pain coping skills training to manage osteoarthritis pain: a randomized controlled trial	Rini et al [16], 2015	Knee osteoarthritis (N=113)	AIMS 2^q^ ASES^r^ PASC^s^ PANAS^t^	PainCOACH^u^ program for 8 weeks. Control group: same program without PainCOACH access.	Significant improvement in self-efficacy and pain reduction in women from the intervention group.

^a^VAS: Visual Analog Scale.

^b^KOOS: Knee Injury and Osteoarthritis Outcome Score.

^c^WOMAC: Western Ontario and McMaster Universities Osteoarthritis Index.

^d^30-CST: 30-second chair stand test.

^e^NRS: Numeric Rating Scale.

^f^IPAQ-SF: International Physical Activity Questionnaire—Short Form.

^g^FSS: Fatigue Severity Scale.

^h^QUIPA: Quality Indicators for Physiotherapy Management of Hip and Knee Osteoarthritis.

^i^EARS: Exercise Adherence Rating Scale.

^j^HADS: Hospital Anxiety and Depression Scale.

^k^TKS: Tampa Kinesiophobia Scale (ή Tampa Scale for Kinesiophobia).

^l^PAR-Q+: Physical Activity Readiness Questionnaire Plus.

^m^IKHOAM: Ibadan Knee/Hip Osteoarthritis Outcome Measure.

^n^PACE: Patient-Centered Assessment and Counseling for Exercise.

^o^AAS: Active Australia Survey.

^p^GROC: Global Rating of Change.

^q^AIMS 2: Arthritis Impact Measurement Scales 2.

^r^ASES: American Shoulder and Elbow Surgeons.

^s^PASC: Pain-Related Anxiety and Avoidance Scale.

^t^PANAS: Positive and Negative Affect Schedule.

^u^PainCOACH: pain management coaching.

### Outcomes of Interest

In this systematic review, the outcomes of interest were pain, strength, and balance in patients with KOA undergoing telerehabilitation. Pain was assessed in all studies using a variety of tools. The WOMAC was one of the most used tools for pain measurement, especially in studies like those by Bennell et al [19], where significant improvements were observed at the 6th- and 12th-month follow-ups. Other pain measurement tools included the VAS and the Numeric Pain Rating Scale (NPRS). Both tools were used in studies by Lawford et al [14] and Azma et al [9], showing that telerehabilitation interventions led to significant pain reduction compared with traditional physiotherapy. In some cases, such as in the study by Rini et al [16], it was found that women in the telerehabilitation group experienced a greater reduction in pain compared with men.

In terms of strength and balance, fewer studies evaluated these outcomes, and the tools used were less consistent. The 30-CST and the TUG tests were used to assess lower body strength and balance. In particular, the study by Tore et al [13] found significant improvements in both strength and balance in the telerehabilitation group compared with the control group. This study was one of the few that specifically measured these outcomes. The improvements in strength and balance in this study highlight the potential benefits of telerehabilitation for enhancing these physical capacities, though such outcomes were not consistently reported across other studies.

## Discussion

### Principal Findings

This systematic review examined 6 RCTs assessing the effectiveness of telerehabilitation for KOA. The findings indicate that telerehabilitation can serve as an effective alternative to conventional rehabilitation, demonstrating improvements in pain management, strength, and balance. However, the extent of these benefits varied across studies, with some showing significant clinical improvements while others reported comparable outcomes between telerehabilitation and in-person rehabilitation.

For instance, Azma et al [9] and Odole et al [6] reported significant pain reduction in both intervention and control groups, suggesting that while telerehabilitation is beneficial, it may not always be superior to conventional rehabilitation. On the other hand, Tore et al [13] found substantial improvements in strength and balance among telerehabilitation participants, highlighting its potential to address key functional limitations in patients with KOA. This aligns with the broader understanding that while telerehabilitation effectively delivers structured rehabilitation, its impact may depend on factors such as intervention format, patient adherence, and baseline severity of KOA.

Compared with previous systematic reviews, such as Xie et al [1], which primarily focused on pain and physical function, our study expands the scope by incorporating strength and balance—2 critical components of functional ability and fall prevention that were previously overlooked. In addition, Xie et al [1] included studies up to April 2020, covering 6 RCTs with 791 patients, whereas this review extends the search to May 2024, adding 6 more RCTs. This expanded dataset strengthens the generalizability of our findings and provides a more comprehensive evaluation of telerehabilitation’s role in KOA management.

### Heterogeneity in Study Design and Outcomes

The included studies exhibited substantial heterogeneity regarding intervention modalities, outcome measures, and patient populations, which limited direct comparisons with a very high homogeneity index (*I*^2^=95%). Some trials, such as Bennell et al [15] and Rini et al [16], incorporated therapist-led coaching, whereas others, like Lawford et al [14], relied on self-guided programs with minimal professional interaction. Pain was assessed using different tools (eg, WOMAC and VAS), strength was measured through varied functional tests (eg, 30-second chair stand test), and balance assessments included diverse protocols (eg, timed up and go test). Such methodological variability complicates the pooling of results, necessitating a narrative synthesis rather than a meta-analysis.

Furthermore, differences in study populations introduced additional variability. Odole et al [6] included relatively younger participants, while Azma et al [9] focused on older adults with advanced KOA. Given that older patients often have greater physical limitations combined with cognitive impairments and reduced exercise adherence, the effectiveness of telerehabilitation may differ across age groups. Future research should conduct subgroup analyses comparing intervention effectiveness across different demographic and disease severity levels to clarify which patients benefit most from telerehabilitation.

### Comparison With Previous Work

Unlike previous systematic reviews, which primarily examined general digital rehabilitation interventions, this review categorizes telerehabilitation strategies based on their mode of delivery, distinguishing between real-time therapist-guided interventions and self-managed programs. This distinction is crucial, as previous findings have been inconsistent due to a lack of differentiation in rehabilitation modalities.

Furthermore, while Xie et al [1] relied solely on the PEDro scale for risk-of-bias assessment, this review uses both the PEDro scale and the Downs and Black checklist, offering a more rigorous evaluation of methodological quality. In addition, a sensitivity analysis was conducted to assess the robustness of findings, an approach not included in previous reviews. These methodological enhancements provide a stronger foundation for evaluating telerehabilitation's effectiveness and guide recommendations for its integration into clinical practice.

### Risk of Bias and Study Quality

Several methodological limitations were identified across the included RCTs. Selection bias was evident in studies such as Lawford et al [14], where randomization procedures were not fully detailed, raising concerns about allocation concealment. Performance bias was also a common issue, as blinding of participants and therapists is inherently difficult in telerehabilitation studies. This could have influenced subjective outcomes such as pain perception. In addition, high attrition rates were noted in studies like that of Rini et al [16], potentially affecting the validity of long-term conclusions.

Despite these limitations, most included studies were classified as fair to good quality. Future research should prioritize strategies to mitigate bias, such as using more robust randomization methods, improving participant retention, and using objective outcome measures where possible.

### Integration of Telerehabilitation Into KOA Management

Telerehabilitation offers multiple advantages, particularly for patients with mobility limitations or geographic barriers to health care access. Its flexibility allows for continuity of care, reducing dependence on in-person visits. However, several challenges remain for its widespread implementation.

One major issue is adherence, as engagement tends to decline in self-managed programs. Bennell et al [15] found that interventions with therapist-guided coaching led to higher adherence rates than fully self-directed programs. This suggests that maintaining patient interaction with health care professionals—even remotely—may be key to optimizing outcomes.

In addition, regulatory and reimbursement barriers limit the accessibility of telerehabilitation in many health care systems. Standardizing reimbursement policies and integrating telerehabilitation into clinical guidelines could enhance adoption. Furthermore, technological barriers, such as digital literacy challenges among older adults, must be addressed through user-friendly platforms and comprehensive patient education programs.

### Strengths and Limitations

A key strength of this review is its inclusion of a broader range of outcome measures beyond pain reduction, allowing for a more holistic assessment of telerehabilitation’s benefits. The extensive literature search across multiple databases minimized selection bias and ensured a thorough synthesis of available evidence. The use of validated quality assessment tools further strengthened methodological rigor.

However, several limitations should be acknowledged. Heterogeneity in intervention protocols and outcome measures limited direct comparisons and precluded meta-analysis. Language bias may also be present, as only English-language studies were included. In addition, most studies had short follow-up periods, making it difficult to determine the long-term sustainability of telerehabilitation outcomes. Future trials should incorporate extended follow-up periods to assess whether improvements in pain, strength, and balance persist over time.

### Future Directions

To refine the evidence for telerehabilitation, future research should focus on the following:

1. Long-term outcomes: conducting longitudinal studies to determine whether benefits persist beyond the immediate post-intervention period.
2. Standardized protocols: developing uniform telerehabilitation protocols to facilitate comparisons across studies.
3. Technology integration: exploring artificial intelligence-driven rehabilitation tools and wearable motion-tracking devices to enhance remote therapy effectiveness.
4. Subgroup analyses: identifying which patient populations (eg, age groups and severity levels) derive the greatest benefit from telerehabilitation.
5. Cost-effectiveness studies: evaluating the economic impact of telerehabilitation compared to traditional rehabilitation models.

### Conclusion

This systematic review highlights the potential of telerehabilitation as a viable alternative to conventional rehabilitation for KOA, demonstrating benefits in pain reduction, strength, and balance. However, its effectiveness varies depending on intervention type, patient engagement, and delivery method. In comparison to previous reviews, this study broadens the scope of analysis, incorporates 6 additional RCTs, applies a dual-method risk-of-bias assessment, and differentiates telerehabilitation modalities.

By distinguishing therapist-guided interventions from self-managed programs, this review provides novel insights into how telerehabilitation can be optimized for KOA management. Addressing current methodological limitations and refining intervention strategies will be crucial in ensuring its long-term success. With further research and policy support, telerehabilitation has the potential to become an integral component of rehabilitation care, improving accessibility and patient outcomes in KOA treatment.

## Appendix

Multimedia Appendix 1 Preferred Reporting Items for Systematic Reviews and Meta-Analyses (PRISMA) checklist.

## Abbreviations

30-CST: 30-second chair stand test
KOA: knee osteoarthritis
NPRS: Numeric Pain Rating Scale
PAINCOACH: pain management coaching
PCST: pain coping skills training
PEDro: Physiotherapy Evidence Database
PICO: Population, Intervention, Comparison, Outcome
PRISMA: Preferred Reporting Items for Systematic Reviews and Meta-Analyses
PROSPERO: International Prospective Register of Systematic Reviews
RCT: randomized controlled trial
TUG: timed up and go test
VAS: Visual Analog Scale
WOMAC: Western Ontario and McMaster Universities Osteoarthritis Index

## References

[1] Xie S, Wang Q, Wang L, Wang L, Song K, He C (2021). Effect of internet-based rehabilitation programs on improvement of pain and physical function in patients with knee osteoarthritis: systematic review and meta-analysis of randomized controlled trials. J Med Internet Res.

[2] Abramoff B, Caldera FE (2020). Osteoarthritis: pathology, diagnosis, and treatment options. Med Clin North Am.

[3] Cui A, Li H, Wang D, Zhong J, Chen Y, Lu H (2020). Global, regional prevalence, incidence and risk factors of knee osteoarthritis in population-based studies. EClinicalMedicine.

[4] Xiang W, Wang JY, Ji BJ, Li LJ, Xiang H (2023). Effectiveness of different telerehabilitation strategies on pain and physical function in patients with knee osteoarthritis: systematic review and meta-analysis. J Med Internet Res.

[5] Kolasinski SL, Neogi T, Hochberg MC, Oatis C, Guyatt G, Block J, Callahan L, Copenhaver C, Dodge C, Felson D, Gellar K, Harvey WF, Hawker G, Herzig E, Kwoh CK, Nelson AE, Samuels J, Scanzello C, White D, Wise B, Altman RD, DiRenzo D, Fontanarosa J, Giradi G, Ishimori M, Misra D, Shah AA, Shmagel AK, Thoma LM, Turgunbaev M, Turner AS, Reston J (2020). 2019 American college of rheumatology/arthritis foundation guideline for the management of osteoarthritis of the hand, hip, and knee. Arthritis Rheumatol.

[6] Odole AC, Ojo OD (2013). A telephone-based physiotherapy intervention for patients with osteoarthritis of the knee. Int J Telerehabil.

[7] Xia B, Chen D, Zhang J, Hu S, Jin H, Tong P (2014). Osteoarthritis pathogenesis: a review of molecular mechanisms. Calcif Tissue Int.

[8] Naeemabadi M, Fazlali H, Najafi S, Dinesen B, Hansen J (2020). Telerehabilitation for patients with knee osteoarthritis: A focused review of technologies and teleservices. JMIR Biomed Eng.

[9] Azma K, RezaSoltani Z, Rezaeimoghaddam F, Dadarkhah A, Mohsenolhosseini S (2018). Efficacy of tele-rehabilitation compared with office-based physical therapy in patients with knee osteoarthritis: A randomized clinical trial. J Telemed Telecare.

[10] Moher D, Liberati A, Tetzlaff J, Altman DG, PRISMA Group (2009). Preferred reporting items for systematic reviews and meta-analyses: the PRISMA statement. BMJ.

[11] Booth A, Clarke M, Dooley G, Ghersi D, Moher D, Petticrew M, Stewart L (2012). The nuts and bolts of PROSPERO: an international prospective register of systematic reviews. Syst Rev.

[12] Guyatt GH, Oxman AD, Kunz R, Atkins D, Brozek J, Vist G, Alderson P, Glasziou P, Falck-Ytter Y, Schünemann HJ (2011). GRADE guidelines: 2. Framing the question and deciding on important outcomes. J Clin Epidemiol.

[13] Tore NG, Oskay D, Haznedaroglu S (2023). The quality of physiotherapy and rehabilitation program and the effect of telerehabilitation on patients with knee osteoarthritis. Clin Rheumatol.

[14] Lawford BJ, Hinman RS, Kasza J, Nelligan R, Keefe F, Rini C, Bennell KL (2018). Moderators of effects of internet-delivered exercise and pain coping skills training for people with knee osteoarthritis: exploratory analysis of the IMPACT randomized controlled trial. J Med Internet Res.

[15] Bennell KL, Campbell PK, Egerton T, Metcalf B, Kasza J, Forbes A, Bills C, Gale J, Harris A, Kolt GS, Bunker SJ, Hunter DJ, Brand CA, Hinman RS (2017). Telephone coaching to enhance a home-based physical activity program for knee osteoarthritis: A randomized clinical trial. Arthritis Care Res (Hoboken).

[16] Rini C, Porter LS, Somers TJ, McKee DC, DeVellis RF, Smith M, Winkel G, Ahern DK, Goldman R, Stiller JL, Mariani C, Patterson C, Jordan JM, Caldwell DS, Keefe FJ (2015). Automated internet-based pain coping skills training to manage osteoarthritis pain: a randomized controlled trial. Pain.

[17] Downs SH, Black N (1998). The feasibility of creating a checklist for the assessment of the methodological quality both of randomised and non-randomised studies of health care interventions. J Epidemiol Community Health.

[18] Cashin AG, McAuley JH (2020). Clinimetrics: physiotherapy evidence database (PEDro) scale. J Physiother.

[19] Bennell K, Nelligan RK, Schwartz S, Kasza J, Kimp A, Crofts SJ, Hinman RS (2020). Behavior change text messages for home exercise adherence in knee osteoarthritis: randomized trial. J Med Internet Res.

